# Waiting more than 24 hours for hip fracture surgery is associated with increased risk of adverse outcomes for sicker patients: a nationwide cohort study of 63,998 patients using the Swedish Hip Fracture Register

**DOI:** 10.2340/17453674.2023.9595

**Published:** 2023-02-27

**Authors:** Katarina GREVE, Stina EK, Erzsébet BARTHA, Karin MODIG, Margareta HEDSTRÖM

**Affiliations:** 1 Department of Clinical Science, Intervention and Technology (CLINTEC), Karolinska Institutet, Stockholm; 2 Function Perioperative Medicine and Intensive Care (PMI), Karolinska University Hospital, Stockholm; 3 Institute of Environmental Medicine (IMM), Karolinska Institutet, Stockholm; 4 Trauma and Reparative Medicine Theme (TRM), Karolinska University Hospital, Stockholm, Sweden

## Abstract

**Background and purpose:**

Waiting time to surgery is a modifiable risk factor in hip fracture surgery. However, there is no consensus regarding the acceptable duration of waiting time. We used the Swedish Hip Fracture Register RIKSHÖFT and 3 administrative registers to explore the association between time to surgery and adverse outcomes after discharge.

**Patients and methods:**

63,998 patients ≥ 65 years, admitted to a hospital between January 1, 2012, and August 31, 2017 were included. Time to surgery was divided into < 12, 12–24, and > 24 hours. Diagnoses investigated were atrial fibrillation/flutter (AF), congestive heart failure (CHF), pneumonia, and “acute ischemia” (a combination of stroke/intracranial bleeding, myocardial infarction, and acute kidney injury). Crude and adjusted survival analyses were performed. Time spent in hospital following the initial hospitalization was described for the 3 groups.

**Results:**

Waiting > 24 hours was associated with an increased risk of AF (HR 1.4, 95%CI 1.2–1.6), CHF (HR 1.3, CI 1.1–1.4) and “acute ischemia” (HR 1.2, CI 1.01–1.3). However, stratifying for ASA grade revealed that these associations were present only in patients with ASA 3–4. There was no association between waiting time and pneumonia after the initial hospitalization (HR 1.1, CI 0.97–1.2), but one was found with pneumonia during hospital stay OR 1.2 (CI 1.1–1.4). Time in hospital after the initial hospitalization was similar over the waiting time groups.

**Conclusion:**

The associations between waiting > 24 hours for hip fracture surgery and AF, CHF, and acute ischemia suggest that shorter waiting time may reduce adverse outcomes for the sicker patients.

Hip fracture is a common and potentially devastating injury that entails a 1-year all-cause mortality of about 25% (1). Waiting time to surgery has been studied as a potential modifiable risk factor of complications and death (2-5), with the suggested mechanism that prolonged waiting exposes the patient to a state of hypercoagulability, catabolism, and stress (6), caused by the injury itself and the immobilization and fasting thereafter (7). Waiting > 24 hours for surgery has also been associated with increased intraoperative instability (8), perhaps partly due to compromised fluid balance.

There is no consensus regarding what duration of waiting time to hip fracture surgery can be considered acceptable. We have previously studied the association between waiting more vs. less than 24 hours to hip fracture surgery and death within 4 months in a large Swedish cohort (9). We did not find an association between waiting longer than 24 hours and an increased risk of mortality among the healthier patients (American Society of Anesthesiologists Physical Status Classification System [ASA] (10) 1 and 2). Among the sicker patients (ASA 3 and 4), there was an association between waiting > 24 hours and death within 4 months, and it was stronger for women than for men. However, evaluating mortality only is insufficient; postoperative morbidity is of importance for quality of life after hip fracture (11).

Therefore, the main objective of this study was to explore the association between waiting time to surgery and adverse outcomes; specifically, atrial fibrillation/flutter, congestive heart failure, pneumonia, and acute ischemia, up to 120 days after initial hospital department discharge. An additional objective was to describe time spent in hospital, after initial hospital department discharge, for patients operated on for hip fracture, based on waiting time to surgery.

## Patients and methods

### Study design

This is a nationwide cohort study of the exposure waiting time to surgery, as registered in the Swedish Hip Fracture Register RIKSHÖFT (SHR) (12). Time to surgery was influenced by nationwide guidelines, which at the time prescribed surgery within 24 hours (13). The outcome “postoperative morbidity” was estimated from diagnoses from the administrative healthcare registers Swedish National Patient Register (NPR) (14) and Swedish Cause of Death Register (CDR) (14). The outcome data is presented for the whole cohort as well as stratified for sex and ASA category and adjusted for potential confounders in 3 different models. The outcome “time readmitted” is constructed from data in NPR. The results are reported as recommended by the The REporting of studies Conducted using Observational Routinely collected health Data (RECORD) Statement (15).

### Setting and participants

SHR is a national clinical quality register on patients with hip fracture. It contains prospective data such as fracture type, time to surgery, surgical method, and ASA classification on the individuals therein, routinely collected in the patients’ care processes, and had > 80% completeness between 2008 and 2017 (16). NPR holds information on all inpatient care and outpatient specialized care (i.e., no primary care data) in Sweden and has shown high levels of validity for many diagnoses (17). For this study, we used inpatient data only. CDR is updated every year and includes information on the cause of death of every person who has died in Sweden (14,18). CDR has been reported as having a high concordance with medical records for cardiovascular causes of death, but generally more uncertainty for older people compared with younger (19). The National Prescribed Drug Register (NPD) holds information on all dispensed prescribed drugs in Sweden, including for nursing home residents (14). Linkage between these registers is facilitated by the personal identity numbers assigned to every person residing within the country.

Inclusion criteria were individuals ≥ 65 years of age with hip fracture admitted to a hospital between January 1, 2012, and August 31, 2017, and treated surgically. Exclusion criteria were time to surgery < 2 hours (assumed as erroneous), or > 7 days, ASA score ≥ 5, pathological fracture, death during the initial hospitalization, and missing data regarding date of surgery, surgical method, or ASA score. If an individual appeared in SHR more than once during the study period, only the first fracture was considered.

### Variables

The exposure was waiting time to surgery (time, in hours, between hospital arrival and start of surgery). The continuous variable was transformed into a categorical variable with 3 potential values, < 12 hours, 12–24 hours, and > 24 hours. Predefined cutoffs for time to surgery have been described as arbitrary (5,20). However, surgery within 24 hours was the national recommendation during the study period as it has been suggested as a point of inflection for death and complications (5). As further reduction in time to surgery has been studied (6), those operated on within 12 hours were studied in a separate group.

The outcomes for specific diagnoses were defined as ICD-10 codes registered in NPR or CDR. We selected diagnoses that are common postoperatively and can reasonably be attributed to the wait for hip fracture surgery: atrial fibrillation/flutter (AF): I48, congestive heart failure (CHF): I50, and bacterial pneumonia or pneumonia with an unknown etiology (combined into the variable “pneumonia”): J13–15, J18. Finally, we combined ischemic stroke/intracranial bleeding: I61–64, myocardial infarction (MI): I21–22, and acute kidney failure: N17 into a variable termed “acute ischemia”. This variable was constructed to reflect the organs most vulnerable to perioperative changes in blood pressure (21). Longer waiting time to hip fracture surgery has previously been shown to be associated with intraoperative hypotension (8). However, hypotension alone cannot fully explain perioperative organ injury, and has been suggested to be a biomarker rather than a direct mediator (22).

**Figure 1:**
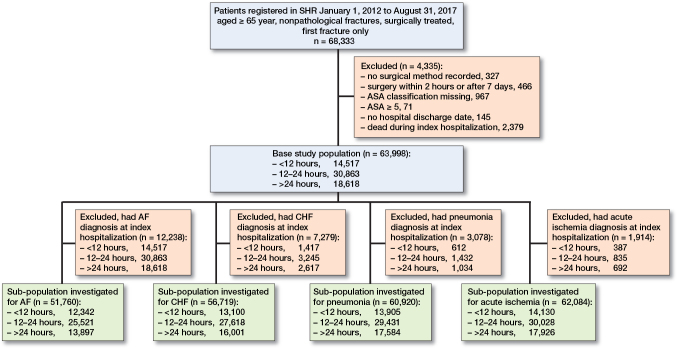
Flowchart of the base study population and the sub-populations.

The diagnoses in NPR are registered at the end of a hospital department stay and it is not possible to determine whether the event occurred before or after the fracture. Due to this, all patients with the outcome diagnoses registered during the index hospitalization were excluded from the analysis of that particular outcome, creating 1 full study population and 4 slightly different sub-populations (Figure 1). If a patient was transferred directly from one department to another, diagnoses originating from the second department could be counted as “outcome diagnoses.” 12,238 patients were excluded for the analysis of AF, 7,279 patients were excluded for the analysis of CHF, 3,078 patients were excluded for the analysis of pneumonia, and 1,914 patients were excluded for the analysis of acute ischemia. Those who died during the index hospitalization were excluded as they were not at-risk for the outcomes. However, because pneumonia has also been reported as one of the most common postoperative complications during the hospitalization for hip fracture (20,23), we additionally performed a regression analysis on the pneumonia cases that occurred during the index hospitalization only, although the potential for reverse causation remains.

The outcome “time spent in hospital after the initial hospitalization” was defined as total nights admitted between the initial hospital department discharge and 120 days, divided into 6 categories: none, < 1 week, 1–2 weeks, 2–3 weeks, 3–4 weeks, and > 4 weeks. Thus, if a patient was transferred to a new department in direct conjuncture with the initial hospitalization, this new hospital stay counted towards “time spent in hospital after the initial hospitalization.” This outcome was selected to reflect differences in overall postoperative morbidity rather than differences in readmission rates. The time categories were chosen for simplicity of interpretation.

ASA classification was used as a measure of comorbidity, and a potential confounder, and was divided into 2 groups (ASA1–2) and (ASA 3–4). A previous diagnosis of each of the outcomes was also considered a potential confounder. NPR was searched for previous registrations of all the outcome diagnoses, except pneumonia, within the last year before the hip fracture. For pneumonia, NPR was searched for registrations within 30 days before the fracture. Diagnoses of dementia (G30–31, F00–03) registered in the NPR in the last 5 years before fracture, were combined with the variable “known dementia” in SHR, creating the composite variable “previous diagnosis of dementia.” Type of surgery was divided into 2 groups, group 1 (intramedullary nail, hemiarthroplasty, and total hip replacement, which are all intramedullary procedures), group 2 (internal fixation other than intramedullary procedures). Antithrombotic therapy was considered a potential confounder. Patients who had filled a prescription for anticoagulants and/or platelet inhibitors, other than aspirin, within 120 days before hospital arrival, were considered to be on antithrombotic therapy. The drugs included are listed in Table 1 (see Appendix).

**Table 1:** ATC codes, generic names, and frequencies (10,359 individuals (16%) out of the base study population of 63,998 individuals had collected a prescription within 120 days before hospital admission) for drugs included in the variable “antithrombotic treatment”

ATC code	Generic drug name	Number of individuals (% of 10,359)
B01AA03	Warfarin	5,557 (54)
B01AA04	Fenprocoumon	9 (0.1)
B01AC04	Clopidogrel	2,312 (22)
B01AC07	Dipyridamole	661 (6)
B01AC22	Prasugrel	4 (0.04)
B01AC24	Ticagrelor	135 (1)
B01AE07	Dabigatran	222 (2)
B01AF01	Rivaroxaban	444 (4)
B01AF02	Apixaban	1,014 (10)
B01AF03	Edoxaban	1 (0.01)

### Statistics

Descriptive baseline characteristics were presented for the whole population and stratified by waiting time to surgery and presented as means, medians, or percentages. Cox proportional hazards models were used to analyze the association between waiting time and occurrence of the outcome variables from initial hospital department discharge until 120 days thereafter. For pneumonia that occurred during the hospitalization, logistic regression was used. A significance level of 95% was chosen and waiting time < 12 hours served as reference group. 3 different models were performed, with Model 1 being crude; Model 2 adjusted for age, sex, and ASA category; and Model 3 adjusted for age, sex, ASA category, type of surgery, previous diagnosis of dementia, previous occurrence of the studied outcome diagnosis, and antithrombotic therapy. The analyses were performed in the whole study sample as well as stratified by sex and ASA category with modified Models 2 and 3.

Differences between the waiting time categories in number of days spent at the hospital after initial discharge up until 120 days were presented with graphs stratified by ASA category.

Statistical analyses were conducted using STATA 16 (2019; StataCorp, College Station, TX, USA), and GraphPad Prism version 9.3.1 (GraphPad Software, CA; https://www.graph-pad.com/).

### Ethics, registration, data sharing plan, funding, and potential conflicts of interest

The study was approved by the regional Ethics Committee of Stockholm, Dnr 2011/1036-31 and 2018/84-32, and was funded by grants provided by the Kamprad Family Foundation for Entrepreneurship, Research and Charity (grant number 20190135), Region Stockholm (ALF project), the Promobilia Foundation, and by Karolinska University Hospital. The funding sources played no role in the investigation. The raw data used in this study is considered sensitive personal information that is protected by Swedish law and cannot therefore be shared without ethical approval. The authors report no conflicts of interest. Completed disclosure forms for this article following the ICMJE template are available on the article page, doi: 10.2340/17453674.2023.9595

## Results

### Descriptive data

63,998 patients were included in the study (Figure 1). Baseline characteristics are given in Table 2. 69% were women, and the mean age was 83 years (SD 8). The overall median waiting time was 20 hours. 23% were operated on within 12 hours, 48% between 12 and 24 hours and 29% after 24 hours. For those operated on after 24 hours, the median waiting time was 32 hours. Overall 30-day mortality was 5% and 120-day mortality was 13%. A majority, 71% of the patients, were admitted from their own home, 19% had a diagnosis of dementia at baseline, and 16% were on antithrombotic therapy.

**Table 2:** Descriptive statistics of study population presented for waiting time to hip fracture surgery less than 12 hours, 12–24 hours, more than 24 hours. Values are number (column %) unless otherwise specified

Factor	< 12 hours	12–24 hours	> 24 hours	Total
Patients (row %)	14,517 (23)	30,863 (48)	18,618 (29)	63,998 (100)
Women	10,146 (70)	21,533 (70)	12,441 (67)	44,120 (69)
Mean age (SD)	83 (8)	83 (8)	83 (8)	83 (8)
ASA 1–2	6,595 (45)	12,779 (41)	6,599 (35)	25,973 (41)
ASA 3–4	7,922 (55)	18,084 (59)	12,019 (65)	38,025 (59)
Cervical hip fracture				
non-displaced, Garden 1+2	2,029 (14)	3,862 (13)	2,312 (12)	8,203 (13)
displaced, Garden 3+4	4,476 (31)	11,982 (39)	7,992 (43)	24,450 (38)
Non-cervical hip fracture	8,012 (55)	15,019 (49)	8,314 (45)	31,345 (49)
Surgical method 1 **^a^**	8,100 (56)	19,211 (62)	11,791 (63)	39,102 (61)
Surgical method 2 **^b^**	6,417 (44)	11,652 (38)	6,827 (37)	24,896 (39)
Hours to surgery, median (IQR)	8 (6–10)	19 (16–21)	32 (27–43)	20 (13–25)
30-day survival (after fracture)	13,810 (95)	29,462 (95)	17,627 (95)	60,899 (95)
4-month survival (after fracture)	12,597 (87)	27,000 (87)	15,931 (86)	55,528 (87)
Admitted from **^c^**				
own home	10,034 (69)	22,233 (72)	13,412 (72)	45,679 (71)
institutional care	3,944 (27)	7,591 (25)	4,429 (24)	15,964 (25)
other	480 (3)	856 (3)	700 (4)	2,036 (3)
On antithrombotic therapy	1,454 (10)	4,341 (14)	4,564 (25)	10,359 (16)
Previously known dementia	3,001 (21)	5,887 (19)	3,272 (18)	12,160 (19)
Previous diagnosis within 1 year of stroke/intracranial bleeding	365 (3)	805 (3)	654 (4)	1,824 (3)
atrial fibrillation/flutter)	1,028 (7)	2,586 (8)	2,403 (13)	6,017 (9)
congestive heart failure	774 (5)	1,789 (6)	1,569 (8)	4,132 (6)
myocardial infarction	159 (1)	368 (1)	336 (2)	863 (1)
acute kidney failure	81 (1)	187 (1)	143 (1)	411 (1)
stroke/intracranial bleeding or				
myocardial infarction or acute				
kidney failure	584 (4)	1,313 (4)	1,089 (6)	2,986 (5)
Previous diagnosis of pneumonia				
(last 30 days)	98 (1)	229 (1)	175 (1)	502 (1)

a Surgical method 1 = intramedullary nail, hemiarthroplasty, or total hip replacement.

b Surgical method 2 = all others, non-intramedullary methods.

c Missing patients in the “admitted from” category = 319 (0.5%).

### Time spent in hospital after initial hospitalization

**Figure 2:**
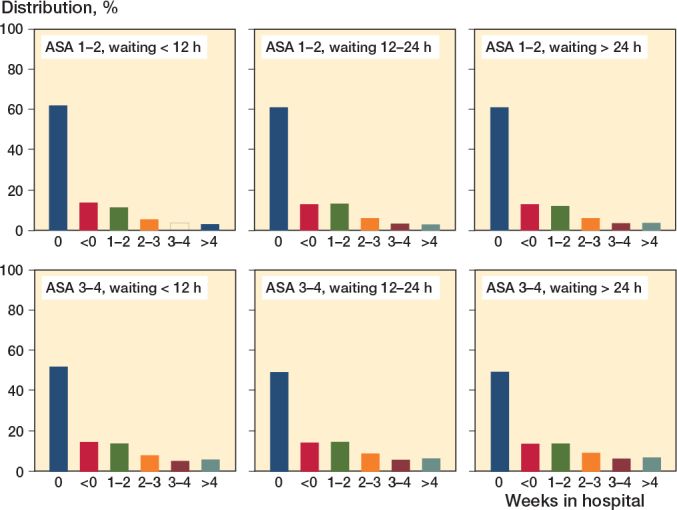
Weeks spent in hospital within 120 days after initial hospital department discharge following hip fracture surgery, stratified by waiting time to surgery category (< 12 hours, 12–24 hours, > 24 hours) and ASA category (1–2 and 3–4 respectively).

There were no major differences in time spent in hospital after the initial hospitalization depending on time to surgery. Approximately 60% of the ASA 1–2 patients and 50% of the ASA 3–4 patients spent no additional time in hospital after discharge from the index hospital department (Figure 2).

### Survival analyses and logistic regression

**Figure 3:**
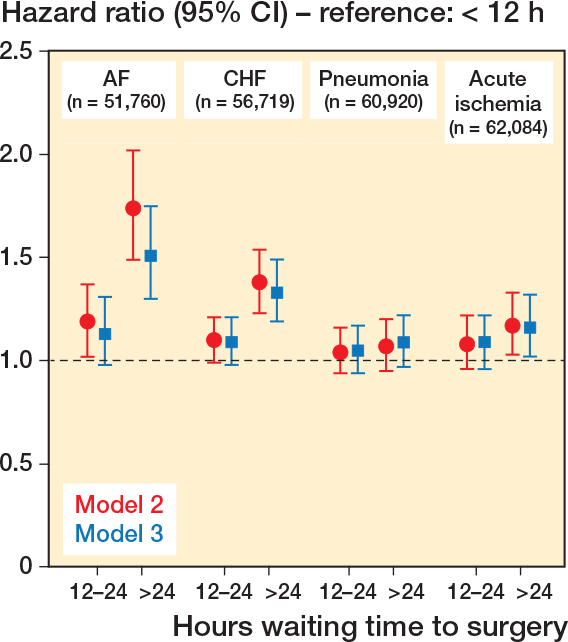
HRs for postoperative morbidity within 120 days of initial hospital department discharge after hip fracture surgery, depending on waiting time to surgery (waiting < 12 hours as reference). Model 2: analyses adjusted for age, sex, and ASA category. Model 3: adjusted for age, sex, ASA category, type of surgery, previous diagnosis of dementia, previous hospitalization for the diagnosis investigated, antithrombotic therapy.

Overall, there was a trend towards longer waiting times being associated with the outcomes investigated (Figure 3), with a similar pattern, albeit with varying effect magnitudes, for all the diagnoses. The differences between those operated on within 12 hours and between 12 and 24 hours were non-significant, but for no outcome was the hazard lower for later surgery compared with earlier.

Hazard ratios and absolute risks for the 4 diagnoses investigated are presented in Table 3. 1,387 patients (3%) had AF within the follow-up period. Survival analyses demonstrated an increased risk of AF in the group operated on after 24 hours compared with those operated on within 12 hours in the fully adjusted model, HR 1.4 (CI 1.2–1.6). Stratifying by sex revealed that the association between waiting > 24 h and AF was similar for women and men, HR 1.4 (CI 1.1–1.7) and HR 1.3 (CI 1.03-1.7), respectively (Table 4, see Appendix). Stratifying by ASA revealed that the associations remained statistically significant for the sicker patients only: HR 1.4 (CI 1.2–1.7) for the ASA 3–4 patients vs. 1.3 (CI 0.98–1.8) for the ASA 1–2 patients (Table 4, see Appendix).

**Table 3:** Association between time to surgery and atrial fibrillation/flutter, congestive heart failure, pneumonia, acute ischemia within 120 days after initial hospital department discharge estimated with Cox proportional hazards regression

Hours t surgery	Cases	Events (%)	Model 1	Model 2	HRs (95% CI) Model 3
AF	51,760	1,387 (3)			
< 12	12,342	250 (2)	Ref.	Ref.	Ref.
12–24	25,521	627 (2)	1.2 (1.1–1.4) **^a^**	1.2 (1.02–1.4) **^a^**	1.1 (0.95–1.3)
> 24	13,897	510 (4)	1.8 (1.6–2.1) **^a^**	1.7 (1.5–2.0) **^a^**	1.4 (1.2–1.6) **^a^**
CHF	56,719	2,484 (4)			
< 12	13,100	483 (4)	Ref.	Ref.	Ref.
12–24	27,618	1,145 (4)	1.1 (1.01–1.3) **^a^**	1.1 (0.99–1.2)	1.1 (0.97–1.2)
> 24	16,001	856 (5)	1.5 (1.3–1.6) **^a^**	1.4 (1.2–1.5) **^a^**	1.3 (1.1–1.4) **^a^**
Pneumonia	60,920	2,226 (4)			
< 12	13,905	473 (3)	Ref.	Ref.	Ref.
12–24	29,431	1,069 (4)	1.1 (0.96–1.2)	1.0 (0.94–1.2)	1.1 (0.94–1.2)
> 24	17,584	684 (4)	1.2 (1.02–1.3) **^a^**	1.1 (0.95–1.2)	1.1 (0.97–1.2)
Acute ischemia	62,084	1,856 (3)			
< 12	14,130	377 (3)	Ref.	Ref.	Ref.
12–24	30,028	888 (3)	1.1 (0.98–1.3)	1.1 (0.96–1.2)	1.1 (0.96–1.2)
> 24	17,926	591 (3)	1.3 (1.1–1.4) **^a^**	1.2 (1.03–1.3) **^a^**	1.2 (1.01–1.3) **^a^**

Model 1: crude.

Model 2: adjusted for age, sex and ASA category.

Model 3: adjusted for age, sex, ASA category, type of surgery, previous diagnosis of dementia, previous diagnosis of the outcome variable, and antithrombotic therapy.

a Statistically significant.

**Table 4:** Association between time to surgery and atrial fibrillation/flutter within 120 days after initial hospital department discharge estimated with Cox proportional hazards regression, sub-population stratified by sex and by ASA category (n = 51,760)

Hours to surgery	Cases	Events (%)	Model 1	HRs (95% CI) Model 2	Model 3
Women **^a^**	36,577	823 (2)			
< 12	8,814	154 (2)	Ref	Ref	Ref
12–24	18,201	374 (2)	1.2 (0.98–1.4)	1.2 (0.96–1.4)	1.1 (0.89–1.3)
> 24	9,562	295 (3)	1.8 (1.5–2.2) **^c^**	1.7 (1.4–2.1) **^c^**	1.4 (1.1–1.7) **^c^**
Men **^a^**	15,183	564 (4)			
< 12	3,528	96 (3)	Ref	Ref	Ref
12–24	7,320	253 (3)	1.3 (1.01–1.6) **^c^**	1.2 (0.97–1.5)	1.1 (0.9–1.4)
> 24	4,335	215 (5)	1.9 (1.5–2.4) **^c^**	1.7 (1.4–2.2) **^c^**	1.3 (1.03–1.7) **^c^**
ASA 1–2 **^b^**	23,526	322 (1)			
< 12	6,108	75 (1)	Ref	Ref	Ref
12–24	11,685	148 (1)	1.0 (0.78–1.4)	1.1 (0.80–1.4)	1.0 (0.77–1.4)
> 24	5,733	99 (2)	1.4 (1.04–1.9) **^c^**	1.5 (1.1–2.0) **^c^**	1.3 (0.98–1.8)
ASA 3–4 **^b^**	28,234	1,065 (4)			
< 12	6,234	175 (3)	Ref	Ref	Ref
12–24	13,836	479 (3)	1.2 (1.04–1.5) **^c^**	1.2 (1.1–1.5) **^c^**	1.1 (0.95–1.3)
> 24	8,164	411 (5)	1.8 (1.5–2.2) **^c^**	1.8 (1.5–2.2) **^c^**	1.4 (1.2–1.7) **^c^**

a Model 1: crude; Model 2: adjusted for age and ASA category; Model 3: adjusted for age, ASA category, type of surgery, previous diagnosis of dementia, previous diagnosis of atrial fibrillation, and antithrombotic therapy.

b Model 1: crude; Model 2: adjusted for age and sex; Model 3: adjusted for age, sex, type of surgery, previous diagnosis of dementia, previous diagnosis of atrial fibrillation, and antithrombotic therapy.

c Statistically significant.

2,484 patients (4%) had CHF within the follow-up period. There was an increased risk of being diagnosed with CHF in the group operated on after 24 hours compared with those operated on within 12 hours in the fully adjusted model, HR 1.3 (CI 1.1–1.4). After stratification by sex, the association remained among women only, HR 1.3 (CI 1.1–1.5) vs. 1.2 (0.99–1.4) for men (Table 5, see Appendix). Similarly to AF, stratifying by ASA grade revealed that an association remained for the sicker patients only, HR 1.3 (CI 1.1–1.4) for ASA 3–4 patients vs. 1.2 (CI 0.95–1.6) for ASA 1–2 patients (Table 5, see Appendix).

**Table 5:** Association between time to surgery and congestive heart failure within 120 days after initial hospital department discharge estimated with Cox proportional hazards regression, sub-population stratified by sex and ASA category (n = 56,719)

Hours to surgery	Cases	Events (%)	Model 1	HRs (95% CI) Model 2	Model 3
Women **^a^**	39,526	1,522 (4)			
< 12	9,227	295 (3)	Ref	Ref	Ref
12–24	19,476	704 (4)	1.1 (0.99–1.3)	1.1 (0.97–1.3)	1.1 (0.95–1.3)
> 24	10,823)	523 (5)	1.5 (1.3–1.8)**^c^**	1.5 (1.3–1.7)**^c^**	1.3 (1.1–1.5)**^c^**
Men **^a^**	17,193	962 (6)			
< 12	3,873	188 (5)	Ref	Ref	Ref
12–24	8,142	441 (5)	1.1 (0.94–1.3)	1.1 (0.90–1.3)	1.1 (0.89–1.5)
> 24	5,178	333 (6)	1.3 (1.12–1.6)**^c^**	1.3 (1.1–1.5)**^c^**	1.2 (0.99–1.4)
ASA 1–2 **^b^**	25,048	494 (2)			
< 12	6,380	114 (2)	Ref	Ref	Ref
12–24	12,348	242 (2)	1.1 (0.89–1.4)	1.1 (0.91–1.4)	1.1 (0.91–1.4)
> 24	6,320	138 (2)	1.2 (0.95–1.6)	1.3 (1.02–1.7)**^c^**	1.2 (0.95–1.6)
ASA 3–4 **^b^**	31,671	1,990 (6)			
< 12	6,720	369 (5)	Ref	Ref	Ref
12–24	15,270	903 (6)	1.1 (0.95–1.2)	1.1 (0.96–1.2)	1.1 (0.94–1.2)
> 24	9,681	718 (7)	1.4 (1.2–1.6)**^c^**	1.4 (1.2–1.6)**^c^**	1.3 (1.1–1.4)**^c^**

a Model 1: crude; Model 2: adjusted for agea and ASA category; Model 3: adjusted for age, ASA category, type of surgery, previous diagnosis of dementia, previous diagnosis of congestive heart failure, and antithrombotic therapy.

b Model 1: crude; Model 2: adjusted for age and sex; Model 3: adjusted for age, sex, type of surgery, previous diagnosis of dementia, previous diagnosis of congestive heart failure, and antithrombotic therapy.

c Statistically significant.

2,226 patients (4%) were registered as having pneumonia within 120 days after discharge. There was an increased risk of pneumonia with longer waiting time in the crude model, but no statistical significance after adjustment, HR 1.1 (CI 0.97–1.2). Similarly, there were no associations between waiting time and pneumonia post index hospitalization in the stratified analyses (Table 6, see Appendix). However, in the analysis of pneumonia cases during the index hospitalization, there was an increased risk of pneumonia among those operated on > 24 hours in the fully adjusted model, OR 1.2 (CI 1.1–1.4) (Table 7, see Appendix).

**Table 6:** Association between time to surgery and pneumonia within 120 days after initial hospital department discharge estimated with Cox proportional hazards regression, sub-population stratified by sex and ASA category (n = 60,920)

Hours to surgery	Cases	Events (%)	Model 1	HRs (95% CI) Model 2	Model 3
Women **^a^**	39,526	1,522 (4)			
< 12	9,227	295 (3)	Ref	Ref	Ref
12–24	19,476	704 (4)	1.1 (0.99–1.3)	1.1 (0.97–1.3)	1.1 (0.95–1.3)
> 24	10,823)	523 (5)	1.5 (1.3–1.8)**^c^**	1.5 (1.3–1.7)**^c^**	1.3 (1.1–1.5)**^c^**
Men **^a^**	17,193	962 (6)			
< 12	3,873	188 (5)	Ref	Ref	Ref
12–24	8,142	441 (5)	1.1 (0.94–1.3)	1.1 (0.90–1.3)	1.1 (0.89–1.5)
> 24	5,178	333 (6)	1.3 (1.12–1.6)**^c^**	1.3 (1.1–1.5)**^c^**	1.2 (0.99–1.4)
ASA 1–2 **^b^**	25,048	494 (2)			
< 12	6,380	114 (2)	Ref	Ref	Ref
12–24	12,348	242 (2)	1.1 (0.89–1.4)	1.1 (0.91–1.4)	1.1 (0.91–1.4)
> 24	6,320	138 (2)	1.2 (0.95–1.6)	1.3 (1.02–1.7)**^c^**	1.2 (0.95–1.6)
ASA 3–4 **^b^**	31,671	1,990 (6)			
< 12	6,720	369 (5)	Ref	Ref	Ref
12–24	15,270	903 (6)	1.1 (0.95–1.2)	1.1 (0.96–1.2)	1.1 (0.94–1.2)
> 24	9,681	718 (7)	1.4 (1.2–1.6)**^c^**	1.4 (1.2–1.6)**^c^**	1.3 (1.1–1.4)**^c^**

a Model 1: crude; Model 2: adjusted for age and ASA category; Model 3: adjusted for age, ASA category, type of surgery, previous diagnosis of dementia, previous diagnosis of pneumonia, and antithrombotic therapy.

b Model 1: crude; Model 2: adjusted for age and sex; Model 3: adjusted for age, sex, type of surgery, previous diagnosis of dementia, previous diagnosis of pneumonia, and antithrombotic therapy.

c Statistically significant.

**Table 7:** Association between time to surgery and pneumonia during the index hospitalization estimated with logistic regression

Hours to surgery	Cases	Events (%)	Model 1	ORs (95% CI) Model 2	Model 3
All	63,998	3,078 (5)			
< 12	14,517	612 (4)	Ref	Ref	Ref
12–24	30,863	1,432 (5)	1.1 (1.0–1.2)	1.1 (0.98–1.2)	1.1 (0.97–1.2)
> 24	18,618	1,034 (6)	1.3 (1.2–1.5) **^a^**	1.3 (1.1–1.4) **^a^**	1.2 (1.1–1.4 **^a^**

Model 1: crude; Model 2: adjusted for age, sex, and ASA category; Model 3: adjusted for age, sex, ASA category, type of surgery, previous diagnosis of dementia, previous diagnosis of pneumonia, and antithrombotic therapy.

a Statistically significant.

For “acute ischemia,” 1,856 patients (3%) were registered with either of the diagnoses that constituted this combined diagnosis during the follow-up period. There was an increased risk of “acute ischemia” for those operated on after 24 hours compared with those operated on within 12 hours in the fully adjusted model, HR 1.2 (CI 1.01–1.3). In contrast to the main analysis, no association between waiting time and acute ischemia remained in the stratified analyses (Table 8, see Appendix), suggesting insufficient statistical power.

**Table 8:** Association between time to surgery and acute ischemia (stroke/intracranial bleeding or myocardial infarction or acute kidney failure) within 120 days after initial hospital department discharge estimated with Cox proportional hazards regression, sub-population stratified by sex and by ASA category (n = 62,084)

Hours to surgery	Cases	Events (%)	Model 1	HRs (95% CI) Model 2	Model 3
Women **^a^**	42,917	1,104 (3)			
< 12	9,898	236 (2)	Ref	Ref	Ref
12–24	21,016	514 (2)	1.0 (0.88–1.2)	1.0 (0.87–1.2)	1.0 (0.86–1.2)
> 24	12,003	354 (3)	1.3 (1.1–1.5) **^c^**	1.2 (1.02–1.4) **^c^**	1.2 (0.99–1.4)
Men **^a^**	19,167	752 (4)			
< 12	4,232	141 (3)	Ref	Ref	Ref
12–24	9,012	374 (4)	1.2 (1.02–1.5) **^c^**	1.2 (0.99–1.5)	1.2 (1.0–1.5)
> 24	5,923	237 (4)	1.2 (0.98–1.5)	1.1 (0.93–1.4)	1.1 (0.92–1.4)
ASA 1–2 **^b^**	25,526	470 (2)			
< 12	6,476	111 (2)	Ref	Ref	Ref
12–24	12,565	225 (2)	1.0 (0.83–1.3)	1.1 (0.84–1.3)	1.1 (0.84–1.3)
> 24	6,485	134 (2)	1.2 (0.94–1.6)	1.2 (0.96–1.6)	1.2 (0.95–1.6)
ASA 3–4 **^b^**	36,558	1,386 (4)			
< 12	7,654	266 (3)	Ref	Ref	Ref
12–24	17,463	663 (4)	1.1 (0.94–1.3)	1.1 (0.95–1.3)	1.1 (0.95–1.3)
> 24	11,441	457 (4)	1.2 (0.99–1.3)	1.2 (0.99–1.3)	1.1 (0.97–1.3)

a Model 1: crude; Model 2: adjusted for age and ASA category; Model 3: adjusted for age, ASA category, type of surgery, previous diagnosis of dementia, previous diagnosis of acute ischemia (stroke/intracranial bleeding or myocardial infarction or acute kidney failure), and antithrombotic therapy.

b Model 1: crude; Model 2: adjusted for age and sex; Model 3: adjusted for age, sex, type of surgery, previous diagnosis of dementia, previous diagnosis of acute ischemia (stroke/intracranial bleeding or myocardial infarction or acute kidney failure), and antithrombotic therapy.

c Statistically significant.

## Discussion

In contrast with much of the previous research on waiting time to surgery, which has often focused on mortality (24), this study contributes information on other adverse outcomes. We found that waiting > 24 hours is associated with increased risk of postoperative morbidity, primarily among sicker patients. This is in line with a previous study by us where we found that longer waiting time was associated with an increased risk of mortality among patients with ASA 3–4 but not among those with ASA 1–2 (9).

There are few randomized controlled trials (RCTs) on this subject. The HIP-ATTACK study (6) compared an accelerated surgery group (median waiting time 6 hours), with a control group (median waiting time 24 hours), with no difference in 90-day mortality or major complications. It did, however, detect decreased instances of delirium and urinary tract infection in the accelerated group. Pincus et al. (5) explored the association between waiting time to surgery with death and medical complications at 30 days in a large, population-based Canadian cohort, using time as a continuous variable in a model of risk-adjusted, restricted cubic splines. They found 24 hours to be an inflection point for death as well as for complications.

In our study, those exposed to the longest waiting time (> 24 hours) still had a comparatively short waiting time (median 32 hours in the base population). This means, though there may be a causal link between longer waiting time and postoperative morbidity, that the associations presented here are likely weaker than they would have been, had those patients waited even longer for surgery. The comparatively short waiting time of the patients in the control group of the HIP-ATTACK trial (6) could perhaps explain the lack of difference regarding mortality and complications there as well. Other important factors to consider are that prolonged waiting time to surgery is associated with longer overall length of hospital stay (20) and, importantly, longer time in immobility, care dependency, and pain due to a non-stabilized fracture. This means that increasing wait times could have implications far beyond the scope of this study.

Our study failed to demonstrate an association between longer waiting time and pneumonia diagnosed after discharge from the index hospital department, which could be due to most cases occurring in the early postoperative period (23).

Regarding time spent in hospital after the initial hospitalization, we were unable to demonstrate any major differences between the waiting time groups. As far as we are aware, time spent as an inpatient following hip fracture surgery has not previously been investigated in a Swedish context and could be a topic for future research.

### Strengths and limitations

This nationwide study has high coverage, ensuring high generalizability for similar populations, and the analyses were adjusted for many of the major known confounding factors. A major strength is that the analyses were adjusted for antithrombotic therapy as this is an important determinant for delayed hip fracture surgery (25). Adjusting for antithrombotic therapy can also, indirectly, entail adjustment for unmeasured comorbidity, especially related to the specific heart diseases included in this study.

The greatest limitation of this study is that the diagnoses registered at the point of discharge from the index hospitalization could not be used, which has likely led to overall weaker associations. However, excluding patients with the outcome diagnosis during the index hospitalization was a necessary step to reduce the risk of reverse causality.

Moreover, we only had access to diagnose codes registered in conjunction with hospital visits and not in specialized outpatient care (or in primary care or nursing homes). This implies that this study likely misses cases of the outcomes. Yet this is not problematic unless these missed cases are different in their waiting time compared with the cases we observed in NPR. The cases included may be on the more severe end of the spectrum, which could even increase the clinical relevance.

We acknowledge that some of the more common and/or feared complications of hip fracture and hip fracture surgery have not been investigated in this study. Urinary tract infection, delirium, and pressure sores were not investigated due to a suspicion that these have a high tendency to be under-reported in hospital discharge notes and thus in NPR. Thromboembolic events, such as deep vein thrombosis (DVT) and pulmonary embolism (PE), were not investigated due to their comparatively low prevalence (23). There is also reason to believe that cases of fatal PE may be misclassified due to a declining number of autopsies in Sweden (26).

Like all observational studies, this study cannot prove causal connections. There may also be a risk of residual confounding. One unaddressed potential confounder is the reason for surgical delay (administrative vs. medical). Despite attempts to minimize the risk of reverse causation, it cannot be eliminated completely, which warrants careful interpretation of the results. Adverse events associated with longer waiting time to surgery may partly be due to factors such as untreated pain, uncontrolled fluid balance, and prolonged fasting, which could decrease the risks of waiting for surgery if addressed appropriately. Differences in preoperative management could thus complicate comparisons of the effect of waiting time across studies.

Uniform criteria for postoperative complications across national registers and participating hospitals could be the best estimates of adverse outcomes and could support research in perioperative medicine. The specificity and sensitivity of diagnosis codes from administrative registers to detect adverse outcomes postoperatively need to be addressed in future trials.

Time spent admitted in a hospital can be difficult to compare across healthcare systems, as it depends on what options there are for assisted living outside hospital, but the internal validity remains unaffected.

### Conclusions

Waiting > 24 hours to hip fracture surgery was associated with an increased risk of AF, CHF, and acute ischemia, primarily among sicker patients. These results suggest that shorter waiting time to surgery may reduce adverse outcomes in patients with ASA 3 and 4. This should be considered when creating new guidelines for the management of patients with hip fracture. Moreover, our findings highlight the importance of considering the health status of the patients when conducting research on prognostic factors.

## Notes

This study was conceived by KG, SE, EB, KM, and MH. SE and KG performed the analyses. All authors contributed to the interpretation of the data. KG wrote the initial draft. All authors contributed to revising the manuscript.

The authors would like to thank RIKSHÖFT for providing data to this study.

Handling co-editors: Cecilia Rogmark and Robin Christensen

*Acta* thanks Matthew Costa and Jan-Erik Gjertsen for help with peer review of this study.
